# Are counter movement jump and isometric mid-thigh pull tests reliable, valid, and sensitive measurement instruments when performed after maximal cardiopulmonary exercise testing? A sex-based analysis in elite athletes

**DOI:** 10.3389/fphys.2025.1663590

**Published:** 2025-09-03

**Authors:** Hari Pojskić, Jesper Schiller, Peter Pagels, Thony Ragnarsson, Anna K. Melin

**Affiliations:** ^1^ Department of Sports Science, Linnaeus University, Kalmar, Sweden; ^2^ Åsidan Health Center, Nyköping, Sweden

**Keywords:** jump performance, strength, relative reliability, absolute reliability, constructive validity, smallest worthwhile change, typical error, test sensitivity

## Abstract

Both the countermovement jump (CMJ) and the isometric mid-thigh pull (IMTP) are frequently used performance tests to monitor neuromuscular fatigue and recovery after exhaustive physical activities. However, to date, neither the reliability nor the validity of the CMJ and IMTP performed after the cardiopulmonary exercise testing (CPET) has been studied. Thus, this study primarily aimed to investigate the intrasession relative and absolute reliability of the CMJ and IMTP when performed after the CPET. Second, the study aimed to examine the discriminative validity of the CMJ and IMTP performed after the CPET by differentiating between elite male and female athletes. Twenty-eight female (26.8 ± 6.6 years) and seventeen male (23.8 ± 3.5 years) elite Swedish athletes voluntarily participated in the study. Protocols included anthropometric measurements, a cycle ergometer-based CPET (i.e., VO_2peak_ test), followed by three maximal test-retest CMJ and IMTP trials. Jump height, peak power, and relative peak power during CMJ and peak force and relative peak force during IMTP testing were analysed. Results showed high relative reliability of the CMJ and IMTP in the total sample (ICC: 0.97 and 0.98) and separately in male (ICC: 0.88 and 0.98) and female (ICC: 0.98 and 0.93) athletes. The good absolute reliability of the CMJ and IMTP was evidenced by low within-subjects test-retest variability (CV_WS_%) and typical measurement error percentage, ranging between 5.7% and 6.5% and 6.3% and 8.9%, respectively. Both the CMJ and IMTP showed good test sensitivity, with the smallest worthwhile change exceeding the typical error. The CMJ’s jump height, relative peak power, and IMTP’s peak force showed a large discriminatory capacity to differentiate between male and female athletes (Cohen’s d = 3.92, 1.80 and 5.14, respectively). However, when the peak force was standardised relative to body mass and lean mass, the differences between sexes diminished. In conclusion, given that the CMJ and IMTP tests demonstrated high reliability and sensitivity following CPET, they could be confidently used as practical tools for monitoring neuromuscular fatigue and recovery, even after exhaustive cardiopulmonary exertion activities. Additionally, the demonstrated discriminative validity in differentiating between male and female athletes further supports their role in sex-specific performance profiling.

## 1 Introduction

Physical abilities such as strength, power, and endurance are key performance indicators in many sports, as they determine an athlete’s fundamental abilities to jump and run efficiently (Cormie et al., 2010; Ishida et al., 2021). For instance, well-developed muscular strength and power strongly correlate with advanced agility and athletes’ jumping and sprinting abilities (Maestroni et al., 2020; Suchomel et al., 2016). Furthermore, strength and power are crucial for executing sport-specific technical skills effectively, such as kicking a soccer ball, performing a jump shot in basketball, serving or spiking in volleyball, executing forehands and backhands in tennis, lifting a barbell in Olympic weightlifting, skating in ice hockey, and similar athletic movements (Pojskic et al., 2018a; Secomb et al., 2024; Spiteri et al., 2014; Thomas et al., 2015a; Thomas et al., 2015b; Wang et al., 2016). Consequently, permanent evaluation of strength and power is crucial for monitoring the efficacy of strength and conditioning programs, athletes’ progression, rehabilitation status, recovery after injury, neuromuscular fatigue and talent identification (de la Motte et al., 2017; Herda, 2022; Suchomel et al., 2016; Tanner and Gore, 2012).

Strength is defined as the ability of a single muscle or muscle group to exert force (Tanner and Gore, 2012). It is commonly assessed dynamically using the one-repetition maximum (1RM), which measures the greatest amount of resistance (i.e., load) that can be moved through the entire range of motion in a controlled manner (Charles et al., 2018). Typical examples include 1RM squat or bench press, both of which require technical proficiency to ensure safety during execution (Comfort et al., 2019). Strength can also be evaluated under static conditions through isometric testing, where muscle tension is generated without visible joint movement, meaning the muscle contracts without changing length, and the limb remains stationary (Comfort et al., 2019). A widely used example in clinical settings is the handgrip strength test, which measures the maximal isometric force of the forearm and hand muscles using a hand dynamometer (Roberts et al., 2011). In contrast, sport performance settings often employ the Isometric Mid-Thigh Pull (IMTP) test to assess whole-body maximal force production. This test involves the athlete pulling upwards on an immovable bar while standing in a position resembling the second pull phase of Olympic lifting. The IMPT is typically performed using a force plate or load cell (i.e., dynamometer) (Comfort et al., 2019). Due to its static nature and simplicity of performance, it is considered a safer and less fatiguing alternative to the 1RM squat (Comfort et al., 2019) and has demonstrated high validity and reliability (De Witt et al., 2018; Drake et al., 2017; Dos’ Santos et al., 2018) correlating with sprinting, jumping and agility performance across various sports (Spiteri et al., 2014; Thomas et al., 2015a; Thomas et al., 2015b; Wang et al., 2016; Townsend et al., 2019).

Power is defined as the product of force and velocity or the amount of work produced per unit of time (Cronin and Sleivert, 2005). The most common test for measuring lower-body power across various sports is the countermovement jump (CMJ) test (Markovic et al., 2004). CMJ is a vertical jump test that utilises the stretch-shortening cycle by requiring the athlete to perform a rapid downward movement followed by an explosive upward jump. It is widely recognized as a reliable and practical tool for assessing lower-body power output, and is commonly performed on a force plate (Carroll et al., 2019; Gathercole et al., 2015; Markovic et al., 2004; Pojskić et al., 2022). Beyond its utility in power assessment, CMJ has demonstrated broad applicability in performance monitoring, talent identification and development, fatigue and neuromuscular status tracking, injury prevention, risk profiling, and return-to-play protocols (Bishop et al., 2021; Hewett et al., 2005; Pojskic et al., 2018a; Pojskic et al., 2018b; Robles-Palazón et al., 2023; Suchomel et al., 2016).

Although both the IMTP and CMJ have demonstrated high reliability across numerous methodological studies (Carroll et al., 2019; Markovic et al., 2004; Pojskić et al., 2022), a discrepancy exists between how scientists evaluate the metric values of physical tests and how coaches perform the tests in the field. In a controlled research context, tests are commonly administered individually, in a non-fatigued state, and following a standardised warm-up protocol. This approach minimises measurement error, as the performance of one test or different warm-up conditions can influence the subsequent test outcomes by either improving or deteriorating performance (Pagaduan et al., 2012; Pojskic et al., 2015; Pojskic et al., 2023; Tanner and Gore, 2012). On the other hand, to save time and costs, coaches often evaluate athletes’ performances through multiple test batteries, where strength, power, and aerobic capacity are assessed in a single testing session using various test orders and training periods (e.g., pre- or post-session) (Scinicarelli et al., 2021). Given these contrasting approaches, it is crucial to investigate whether the reliability metrics of the IMTP and CMJ remain robust with high-reliability metrics when performed consecutively in a fatigued state, such as immediately following a cardiopulmonary exercise testing (CPET), a maximum effort protocol performed to exhaustion. Such research would provide valuable insight into the ecological validity of these tests in real-world performance monitoring scenarios.

The CPET is a widely used and objective method for evaluating the cardiovascular, pulmonary, and skeletal muscle capacities under exercise-induced stress (Tran, 2018). It is commonly employed in research, sporting environments, and laboratory settings. Exercise stress is usually induced by running on a treadmill or cycling on a cycle ergometer, while constantly measuring oxygen consumption, with VO_2_max as one of the primary variables. The tests are usually performed incrementally with gradual increases in speed, inclination and resistance. The induced exercise stress, intensity and fatigue during CPET are usually evaluated simultaneously by several objective and subjective methods. For instance, blood lactate levels are measured using a finger stick capillary blood sampling method as one of the simplest methods for evaluating exercise intensity. During CPET, blood lactate levels rise gradually until reaching a point called the Onset of Blood Lactate Accumulation, which corresponds to 4 mmol/L. Beyond this point, blood lactate production exceeds clearance, leading to accumulation in the blood and increased acidity in the muscles, which can impair muscle function and contribute to the sensation of fatigue (Goodwin et al., 2007). Research indicates that blood lactate levels in athletes tend to return to near-baseline levels (approximately 1–2 mmol/L) within 30–60 min after a maximal CPET (Hinojosa et al., 2021; Katoch et al., 2025). Therefore, any test conducted within 30 min following a can be regarded as taking place during a state of elevated blood lactate, which is associated with fatigue. Another objective measure of physical exertion during CPET is the direct and constant measurement of heart rate (HR), as HR and workload have a close and positive linear relationship (Charles et al., 2018). For subjective evaluation of perceived exercise strain, several scales, such as the Borg Rate of Perceived Exertion Scale (RPE) and the Borg category rating scale (CR-10), were developed (Borg, 1982; Noble et al., 1983; Charles et al., 2018). The ratings of RPE have been shown to be linearly related to increased exercise intensity (Noble et al., 1983) and highly correlated with HR, while the CR-10 is more suitable for determining breathing difficulties, aches, and pain (Borg, 1982). These validated scales are useful as indicators of training intensity and impending fatigue in exercise testing (Dantas et al., 2015; Charles et al., 2018).

Since the execution of one test can affect the result of the following test (Tanner and Gore, 2012), it is essential to evaluate whether a test provides trustworthy and meaningful results when performed consecutively in contextual settings, such as match play or competition, particularly when athletes experience acute fatigue. To achieve this, the test is evaluated for its relative and absolute reliability, sensitivity, and validity, thereby enhancing both scientific rigour and practical application. Relative reliability refers to the extent to which individuals maintain their position or ranking within a group across repeated test trials, and it is commonly quantified using the Intraclass Correlation Coefficient (ICC) (Koo and Li, 2016; Weir, 2005). In contrast, absolute reliability measures the degree of variation in repeated scores for an individual, typically expressed in the same units as the test or as a coefficient of variation (CV) between individuals' test-retest trials (Hopkins, 2000). Test sensitivity refers to a test’s ability to detect meaningful changes in performance. A test with high sensitivity will have a small typical (measurement) error relative to the expected performance changes. This concept is closely linked to the smallest worthwhile change (SWC), which refers to the smallest change in a score that is considered beneficial or meaningful. If the typical error exceeds the SWC, the test may lack the sensitivity to detect small but significant improvements (Hopkins, 2004). Given that a test can be highly reliable but still invalid, it is also essential to determine test validity (Gratton and Jones, 2010). One way is to evaluate a test’s discriminative validity, which is a subtype of construct validity that refers to the ability of a test to distinguish between different constructs or different groups that should theoretically not be related or equivalent (e.g., male and female athletes, elite and sub-elite athletes) (Thomas et al., 2015c; Pojskic et al., 2020; Pojskic et al., 2019; Pojskic et al., 2018b).

Moreover, women are significantly underrepresented in exercise science (Costello et al., 2014; Mujika and Taipale, 2019), which usually leads to research based on men being applied to women, despite performance differences between the sexes in elite athletes ranging from 8% to 10% (Sandbakk et al., 2018). Consequently, it is relevant to evaluate physical capacities separately in men and women. Furthermore, there is a lack of standardised test protocols for assessing physical abilities when comparing athletes from different sports and sex groups in studies for various reasons. Administering simple and practical test protocols that do not require a high level of skill proficiency and familiarisation, such as CMJ, IMTP, and cycle-ergometer CPET, could enable performance evaluation and comparison in heterogeneous groups of athletes and research samples. Given that no study has been published to date that has examined the reliability and validity of the CMJ and IMTP tests when performed following exhaustive cardiopulmonary exertion activities, there are limited reliability metrics (i.e., ICC, CV, TE, SWC) available in this research design context, highlighting a gap in the existing literature.

Therefore, the primary objective of this study was to determine whether the CMJ and IMTP tests are reliable and sensitive measurement instruments when performed after CPET in a heterogeneous group of elite athletes. A secondary aim was to examine the reliability metrics separately for male and female athletes. The third aim was to explore the discriminative validity of the CMJ and IMTP by differentiating men and women. Based on the previous studies, we hypothesised that both CMJ and IMTP would demonstrate high reliability and discriminatory power following CPET.

## 2 Materials and methods

### 2.1 Experimental design

**FIGURE 1:**
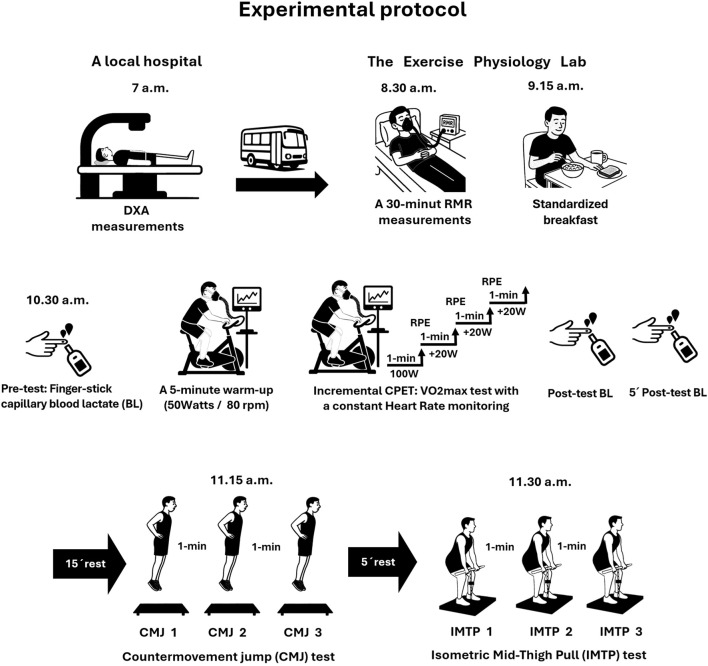
Experimental protocol flowchart. DXA = dual-energy X-ray absorptiometry; RMR = resting metabolic rate; CPET = cardiopulmonary exercise testing; RPE = rate of perceived exertion; rpm = revolutions per minute.

This method study was part of a larger research project, the Relative Energy Deficiency in Sport (REDs) Swedish study. This part of the study was performed in a laboratory setting at the Linnaeus University. It consisted of anthropometric measurements, resting metabolic rate measurements, a cycle ergometer-based CPET (i.e., VO_2peak_ test), followed by CMJ and IMTP tests (see Figure 1 Experimental Protocol). In the final phase, reliability analysis was conducted through the intrasession test-retest measurements of the CMJ and IMTP tests.

### 2.2 Participants

Twenty-eight female and seventeen male elite athletes voluntarily participated in the study (Table 1). They were recruited through an initial on-line survey study of the REDs Sweden project, which included athletes from Swedish National team sports representing ball games (n = 15), weight category (n = 7), technical (n = 3), aesthetic (n = 5), strength (n = 4) and endurance (n = 11) sports. All participants were healthy and free of current injuries or neuromuscular or chronic diseases (e.g., diabetes or hypothyroidism). Since this method study was part of a larger research project investigating various aspects (e.g., nutritional status, microbiota, and performance aspects) of REDs among Swedish elite athletes, only female athletes not using hormonal contraceptives were included in the study. Systematic reviews have reported no or trivial alterations of strength-related measures by the fluctuations in ovarian sex hormones during the menstrual cycle (Blagrove et al., 2020; McNulty et al., 2020; Colenso-Semple et al., 2023). However, since the study also evaluated physiological aspects among eumenorrheic and amenorrhoeic athletes that might fluctuate during the menstrual cycle, such as body weight (Desbrow et al., 2019), resting metabolic rate (RMR) (Benton et al., 2020), we chose to test eumenorrheic athletes in the early follicular phase, where oestrogen and progesterone levels are low (Elliot-Sale et al., 2021). Hence, menstruating athletes were assessed on days 2–5 after the first day of menstrual bleeding, while amenorrhoeic athletes were assessed when suited. Participants were asked to refrain from high-intensity training for at least 24 h before testing. Before commencing the study, participants were informed both orally and in writing about the study design, protocols, benefits, potential risks, and their right to withdraw without explanation. They provided signed informed consent. The study was conducted in accordance with the Helsinki Declaration and was approved by the Regional Ethical Committee in Medicine (Dnr: 2021-07031-01).

**TABLE 1:** Descriptive statistics and differences between male and female athletes in age, anthropometrics, and physiological and performance-related variables.

Variables	Total			Women (n = 28)			Men (n = 17)			t-test value	p-value	ES
	Mean ± SD	CV%	Min–Max	Mean ± SD	CV%	Min–Max	Mean ± SD	CV%	Min–Max			
Age (years)	25.7 ± 5.8	22.6	19–43	26.8 ± 6.6	24.7	19–43	23.8 ± 3.5	14.7	19–32	2.00	0.02	0.54
Body height (m)	174.2 ± 8.9	5.1	151.0–192.0	170.0 ± 7.4	4.4	151.0–184.0	181.0 ± 6.9	3.8	168.0–192.0	4.91	0.00	1.51
Body weight (kg)	70.5 ± 13.0	18.5	49.5–99.6	64.2 ± 10.2	15.9	49.5–92.0	80.9 ± 10.5	12.9	59.0–99.6	5.25	0.00	1.62
BMI (kg/m^2^)	23.1 ± 2.7	11.5	18.4–31.5	22.2 ± 2.7	12.0	18.4–31.46	24.7 ± 1.9	7.7	19.9–27.4	3.35	0.00	1.03
Fat (%)	19.7 ± 6.5	33.2	9.30–41.8	22.4 ± 5.9	26.1	12.7–41.8	15.3 ± 5.1	33.4	9.3–28.0	4.16	0.00	1.28
Lean body mass (%)	77.2 ± 6.4	8.3	56.1–87.1	74.4 ± 5.7	7.7	50.6–83.7	81.7 ± 4.7	5.7	70.2–87.1	4.38	0.00	1.35
VO_2peak_ (mL/kg/min)	49.3 ± 7.4	15.0	35.0–67.3	48.1 ± 7.4	15.3	35.0–62.8	51.4 ± 7.1	13.9	42.0–67.3	1.44	0.08	0.44
VO_2peak_ (L/min)	3.44 ± 0.7	19.5	2.06–4.8	3.05 ± 0.5	15.9	2.06–4.0	4.08 ± 0.4	9.2	3.56–4.81	7.49	0.00	2.30
HR_max_ (beats/min)	181.9 ± 9.3	5.1	158–203	180.2 ± 7.7	4.3	158–191	184.7 ± 11.2	6.1	165–203	1.56	0.06	0.48
BL pre (mmol/L)	1.9 ± 0.6	34.0	0.9–3.7	2.16 ± 0.8	35.2	0.9–3.7	1.7 ± 0.4	24.2	1.1–2.6	2.12	0.02	0.68
BL post (mmol/L)	12.8 ± 2.3	17.9	8.6–17.3	12.5 ± 2.2	17.8	8.6–16.9	13.5 ± 2.4	17.5	10.6–17.3	1.38	0.08	0.44
BL post 5 (mmol/L)	11.3 ± 3.2	28.4	6.1–20.2	10.7 ± 3.2	30.2	6.1–20.2	12.5 ± 3.0	24.1	8.1–17.4	1.74	0.04	0.57
CMJ height (cm)	31.7 ± 8.5	26.5	15.1–54.3	28.7 ± 7.7	26.9	15.1–49.2	37.6 ± 6.7	17.9	28.2–54.3	3.92	0.00	1.21
CMJ Peak Power (W)	3101.3 ± 843.1	27.2	1716.1–4676.9	2959.4 ± 751.9	25.4	1716.1–4325.3	3335.1 ± 952.6	28.6	2015.4–4676.9	1.47	0.07	0.45
R-CMJ Peak Power (W/kg)	43.6 ± 6.5	15.0	32.4–59.2	42.2 ± 6.4	15.1	32.4–57.8	45.8 ± 6.4	14.0	35.2–59.2	1.80	0.04	0.55
IMTP Peak Force (N)	1631.1 ± 477.1	29.2	885.3–2936.1	1382.2 ± 236.7	17.1	885.3–1867.2	2041.1 ± 494.3	24.2	1300.0–2936.1	5.14	0.00	1.85
R-IMTP force (N/kg)	24.7 ± 11.6	47.0	16.5–95.2	25.3 ± 14.1	55.7	16.6–95.2	23.5 ± 5.5	23.3	16.5–37.2	0.51	0.30	0.16
R-Lean Mass IMTP force (N/kg)	29.9 ± 5.2	17.4	20.2–45.5	29.3 ± 4.9	16.7	20.2–41.2	30.8 ± 5.8	18.8	21.4–45.5	0.95	0.17	0.29

BP, blood pressure; SD, standard deviation; CV%, coefficient of variation; Min, minimal values; Max, maximal values; ES, Cohen’s *d* effect size; BMI, body mass index; CMJ, countermovement jump; IMTPT, Isometric mid-thigh pull test; HR_max_, maximal heart rate achieved during VO2max protocol; BL, pre, blood lactate level measured before VO2max protocol; BL, post, blood lactate level measured immediately after VO2max protocol; BL, post 5, blood lactate level measured 5 min after VO2max protocol; R, relative values.

### 2.3 Procedures and measures

Participants were asked to arrive at the local hospital at 7:00 a.m. in a fasted state to undergo a total-body DXA scan. The DXA (Lunar iDXA, EnCore v18.10.105) was used to measure participants’ body mass (BM), body height (BH), and body composition (e.g., fat and lean body mass percentage). The standardised procedure was performed as previously described in the literature (Nana et al., 2015). Upon arrival at the exercise physiology lab around 8 a.m., indirect calorimetry was performed using a canopy hood system (Vyntus CPX, CareFusion, Hochberg, Germany) to measure RMR as described elsewhere (Torstveit et al., 2018). Participants rested for 15 min before the actual 30-min measurement period began, during which the last 20 min were used for RMR analysis. Afterwards, the participants were provided with a standardised breakfast and were additionally informed about the procedures for the CPET, CMJ, and IMTP measurements. The standardised breakfast consisted of either 550 kcal (for participants <70 kg BW: 70 g CHO, 25 g protein, 20 g fat) or 800 kcal (100 g CHO, 35 g protein, 30 g fat).

#### 2.3.1 CPET measurements

Prior to the CPET measurements, participants were asked to sit on the electrically braked cycle-ergometer (Monark LC7TT, Monark Exercise AB, Vansbro, Sweden) and adjust the saddle and handlebar height and distance. This enhanced the validity and reproducibility of the measurements and comfort, allowing participants to focus fully on the test and achieve their true performance capacity. Then, an HR monitor (Polar RS400; Polar, Kempele, Finland) and an Oro-nasal face mask (7450 Series V2, The Hans Rudolph Inc., Kansas, United States) were mounted, and the first out of three capillary blood lactate samples was collected using a portable, finger-stick blood lactate analyser (The Lactate Plus, Nova Biomedical, Massachusetts, United States). Afterwards, participants proceeded to a standardised warm-up which consisted of 5 min of paddling at a power output of 50 W (W) and an approximate cadence of 80 revolutions per minute.

Immediately after the warm-up, participants continued with the incremental CPET protocol, which consisted of continuously increasing resistance by 20 W each 1-min interval, starting with 100 W in the first interval. During the test, oxygen consumption (VO_2_) and carbon dioxide production (VCO_2_) were constantly measured using a calibrated breath-by-breath gas analysis system (Vyntus CPX, CareFusion, Hochberg, Germany) according to standard laboratory procedures. The RPE and CR-10 scales were used for the subjective evaluation of perceived exercise strain until participants reached levels 18 and 9, respectively. Then, participants continued to pedal without reporting the exercise exertion to stay focused on the test. Participants were asked to pedal, keeping the cadence between 70 and 80 revolutions per minute, until they reached volitional exhaustion, that is, the point at which they could no longer maintain the required workload.

The test was terminated earlier if one of the following criteria for reaching VO_2peak_ was met: (a) levelling of VO_2_ consumption and HR despite increased workload, (b) five beats from maximum HR, (c) a respiratory equivalent ratio above 1.10, (d) RPE < 18 and CR-10 < 8, and/or (e) inability to keep a cadence of 70 revolutions per minute (Denham et al., 2020). The average of the two highest VO_2_ data points was used to determine VO_2peak._ The second and third blood lactate sampling were performed immediately after the CPET and 5 min after. Both the warm-up and CPET were performed on the electrically braked cycle-ergometer for several reasons. Firstly, participants do not require long and specific familiarisation with the cycling technique; therefore, it provides similar testing conditions in a heterogeneous group of athletes. Secondly, it provides an accurate increase in power output ranging between 4 and 1,400 W, relatively independent of pedal cadence (Tanner and Gore, 2012).

#### 2.3.2 CMJ and IMTP measurements

After the CPET protocol and before CMJ and IMTP tests, participants were allowed a 15-min rest period, which they used to rehydrate, eat a fruit snack and recover. The CMJ test was performed using the MuscleLab dual force plate system (Egotest Technology AS, Stathelle, Norway) to assess lower body peak power (W), relative peak power (W/kg) and jump height (m). Participants were instructed to stand with their feet on the two force plates, ensuring each foot was placed on a designated plate and to place their hands on their hips throughout the test to minimise arm involvement and isolate the contribution of the lower body. They were asked not to bend their knees and hips when airborne, but just upon landing on the force plates to provide accurate measurement and reduce the risk of injury. Following a brief warm-up and familiarisation that included three to four submaximal jumps, participants initiated a countermovement by flexing their knees to a comfortable depth and immediately executed an explosive vertical jump. Each participant performed three maximal attempts and was given a 1-min rest period between attempts to ensure maximal effort during each attempt. The test showed high test-retest reliability (ICC > 0.90) in numerous method studies (Carroll et al., 2019; Markovic et al., 2004; Pojskić et al., 2022).

The IMTP test was conducted to assess maximal force output (N) and relative force output (N/kg) during a static position that simulates the first phase of the clean (i.e., the Olympic weightlifting exercise), known as the pull phase or initial pull (De Witt et al., 2018). Participants were positioned in a hip-width stance, with the feet flat on a custom-built wooden platform (80 × 50 cm). Knee-flexion of 140°–145° and hip-flexion of 145°–150° were controlled with a handheld goniometer before each attempt. They were asked to position a solid metal bar (length 80 cm, diameter: 3 cm) at mid-thigh height, simulating the first phase of the clean. The hand grip on the bar was slightly wider than shoulder-width. On one side, the MuscleLab load cell (Egotest Technology AS, Stathelle, Norway) was attached to the bar, while the other was connected via a chain to the platform. The chain and carabiner chain lock allowed the bar to be fixed at a designated height above the floor, adapting to the participant’s required starting position. The participants were instructed to create a pretension that engaged all major muscle groups to avoid slack in the body and jerky movement prior to the pull. Then, upon the command: “3, 2, 1 pull”, participants were asked to exert maximal pulling force straight up against the bar for 3 s, generating force by the legs and hips (i.e., knee and hip extension). To establish a better hand grip, participants used lifting straps. Participants were not allowed to lean their bodies backwards and touch their hips with the bar. Familiarisation and warm-up (i.e., 2% × 50%, 2% × 70%, and once 85%–90% of self-estimated maximal effort) were employed before three maximal attempts, separated by a 1-min rest between each attempt. The test showed high test-retest reliability (ICC > 0.90) in previous studies (De Witt et al., 2018; Dos’ Santos et al., 2018; Drake et al., 2017). All participants were verbally encouraged throughout the testing process. All measurements were conducted under standard laboratory conditions at 21 °C–22 °C, which were permanently monitored.

### 2.4 Statistical analyses

The sample size was estimated *a priori* using a hypothesis testing approach (Arifin, 2018) and a web-based sample size calculator accessible at https://wnarifin.github.io/ssc/ssalpha.html, considering previously published interclass correlation coefficients of CMJ and IMTP (Springham et al., 2024; Bright et al., 2023; Montoro-Bombú et al., 2023; Pichardo et al., 2024). For a minimum acceptable ICC of 0.70, an expected ICC of 0.85, a significance level of 0.05, a statistical power of 0.80, three test attempts and an expected dropout rate of 10%, it was estimated that 42 participants would provide an appropriate sample size.

Descriptive statistics [mean, standard deviation (SD), minimum, and maximum] were calculated for all variables. The Shapiro-Wilk test and visual observation of the normality QQ plots were used to explore a normal distribution of outcome variables. Systematic measurement error of CMJ and IMPT and differences between BL periods (pre, 1- and 5-min post) were evaluated using repeated measures ANOVA. When statistically significant differences were detected between trials for the ANOVA, pairwise comparisons were performed using a Bonferroni *post hoc* test.

Absolute reliability (within-subject variation) was established using the coefficient of variation expressed as a percentage (CV%) according to the following formula: mean value of the trials/TE × 100, where TE (typical error) was calculated by dividing the SD of the trial-to-trial difference score by √2 (Hopkins, 2000). A TE% and CV% of <10% indicated low measurement error and good absolute reliability, respectively (Gathercole et al., 2015; Schambra et al., 2015). The ICC estimates and their 95% confidence intervals (CIs) were calculated based on mean measurements (k = 3 for CMJ and IMTP), absolute agreement, and a two-way mixed-effects model (Weir, 2005). An ICC > 0.70 reflected high (DeVellis and Thorpe, 2021) and above 0.90 excellent (Koo and Li, 2016) relative reliability.

Sensitivity was computed by comparing TE with the SWC, both expressed in the test scores for each test (Hopkins, 2000; Buchheit et al., 2014). The SWC was derived from the between-subject SD multiplied by either 0.2 (SWC_0.2_) (Hopkins, 2004; Pyne et al., 2005), which is the typical small magnitude effect, or 0.5 (SWC_0.5),_ which is an alternate moderate effect (Cohen, 2013). A TE below SWC indicated test sensitivity to be “good”, and a similar TE as SWC was rated “acceptable”. If TE was higher than SWC, it was deemed to have “marginal” sensitivity (Hopkins, 2004; Pyne et al., 2005).

Construct validity was evidenced by differentiating the male and female athletes using an independent t-test. Additionally, magnitude-based effect size with 95% Confidence Intervals (CIs) was calculated, and the following criteria were used: ≤0.2 = trivial, >0.2–0.6 = small, >0.6–1.2 = moderate, >1.2–2.0 = large, and >2.0 very large effect size (Hopkins, 2000). All statistical analyses were performed with SPSS® for Windows (version 30; IBM Corporation, Armonk, NY, United States) and MS Excel charts.

## 3 Results

### 3.1 Descriptive statistics

Descriptive statistics and differences between male and female athletes in terms of age, anthropometrics, physiology, and performance-related variables are presented in Table 1. The results showed big variability (i.e., heterogeneity) in the sample with between-subjects CV > 10%. The female athletes showed higher variability in age and all anthropometric, physiological, and performance variables, except for body fat percentage and CMJ Peak Power. The male athletes were younger, taller, heavier, and had a lower body fat percentage, as well as better absolute values in performance tests, compared to female athletes. Although higher in male (184.7 ± 11.2 beats/minute) athletes, HR max did not show significant differences compared with female (180.2 ± 7.7 beats/minute) athletes. Repeated measures ANOVA revealed a significant effect of time of blood lactate measurements (Wilks’ Lambda: F = 332.610, p < 0.001 and ηp^2^ = 0.953) where pre (1.9 ± 0.6 mmol/L), 1-min post (12.8 ± 2.3 mmol/L) and 5-min post blood lactate (11.3 ± 3.2 mmol/L) levels were significantly different between each other. The visual observation of the normality QQ plots and the Shapiro-Wilk test showed that CMJ and IMTP data for all attempts, when averaged, were found to be normally distributed (p > 0.05).

### 3.2 Reliability and sensitivity of CMJ and IMTP

Descriptive, reliability and sensitivity data for the CMJ and IMTP tests are presented in Table 2. Repeated measures ANOVA showed no significant effect of time, neither for the CMJ nor for IMPT, in three test-retest trials (Wilks’ Lambda: F = 1.032, p = 0.365, ηp^2^ = 0.048; F = 3.204, p = 0.051, ηp^2^ = 0.138, respectively), for the total sample. Similar results are observed in the group of male athletes for CMJ (F = 0.007, p = 0.993, and ηp^2^ = 0.001) and IMTP (F = 0.051, p = 0.951, and ηp^2^ = 0.008). In the group of female athletes, a significant effect of time was observed for test-retest trials in CMJ (F = 3.828, p = 0.035, ηp^2^ = 0.227) and IMTP (F = 6.631, p = 0.005, ηp^2^ = 0.347). Bonferroni *post hoc* analysis showed the first CMJ attempt (28.1 ± 8.1 cm) was significantly lower than the second attempt (28.9 ± 7.8 cm). The first (1,342.9 ± 248.3 N) and second attempts (1,367.8 ± 220.8 N) were statistically lower than the third attempt (1,455.9 ± 259.1) in IMTP.

**TABLE 2:** Descriptive and reliability parameters for CMJ and IMTP for the total sample (n = 45) and separately for male (n = 17) and female (n = 28) athletes.

Paricipants	Variables	Mean ± SD	Min – Max		CV%_(BS)_	CV%_(WS)_	SWC_(0.2)_	SWC_(0.5)_	TE	TE%	Sensitivity	ICC (CI95%)
Total sample	CMJ (cm)	31.7 ± 8.5	15.1	54.3	25.1	5.7	1.6	3.9	2.2	6.9	GOOD	0.97 (0.95–0.98)
	Trial 1	31.2 ± 8.5	14.1	52.6								
	Trial 2	31.7 ± 8.1	14.8	49.2								
	Trial 3	32.2 ± 8.7	16.5	56.1								
	IMTP (N)	1,631.1 ± 477.1	885.3	2,936.1	29.2	6.3	95.3	238.6	128.8	7.9	GOOD	0.98 (0.97–0.99)
	Trial 1	1,601.9 ± 496.1	770.8	2,850.5								
	Trial 2	1,626.7 ± 488.7	798.6	2,915.4								
	Trial 3	1,665.8 ± 473.3	1,086.5	3,042.5								
Male athletes	CMJ (cm)	37.6 ± 6.7	28.2	54.3	17.8	6.1	1.4	3.4	2.6	6.9	GOOD	0.88 (0.70–0.96)
	Trial 1	36.7 ± 6.2	27.9	52.6								
	Trial 2	36.4 ± 6.3	27.2	49.2								
	Trial 3	37.4 ± 7.7	23.4	56.1								
	IMTP (N)	2041.1 ± 494.3	1,300.0	2,936.1	24.2	6.5	98.9	247.1	128.8	6.3	GOOD	0.98 (0.95–0.99)
	Trial 1	2028.6 ± 511.8	1,211.0	2,850.5								
	Trial 2	2052.8 ± 513.9	1,168.6	2,915.4								
	Trial 3	2046.2 ± 514.8	1,200.0	3,042.5								
Female athletes	CMJ (cm)	28.7 ± 7.7	15.1	49.2	26.8	5.9	1.5	3.8	2.3	8.0	GOOD	0.98 (0.96–0.99)
	Trial 1	28.1 ± 8.1^†^	14.1	50.2								
	Trial 2	28.9 ± 7.8	14.8	48.6								
	Trial 3	29.0 ± 7.7	16.5	48.7								
	IMTP (N)	1,382.6 ± 236.7	885.3	1,867.2	17.1	6.2	47.3	118.4	122.5	8.9	MARGINAL	0.93 (0.88–0.97)
	Trial 1	1,342.9 ± 248.3*	770.8	1744.1								
	Trial 2	1,367.8 ± 220.8*	798.6	1,655.8								
	Trial 3	1,455.9 ± 259.1	1,086.5	2,201.6								

CMJ, countermovement jump; IMTP, Isometric mid-thigh pull test; SD, standard deviation; Min, minimum value; Max, maximum value; CV%_(BS)_, *between-subjects* coefficient of variation; CV%_(WS)_, *within-subjects* coefficient of variation; ICC, intraclass correlation coefficient; SWC, smallest worthwhile change; TE, typical error of measurement; CI95%. 95% confidence interval; †, statistically different from the second Trial; *, statistically different from the third Trial.

The absolute reliability was good in the total sample and separately for the male and female athletes. The reliability of the CMJ was better than that of the IMTP, with CV values ranging between 5.7% and 6.1% and 6.3% and 6.5%, respectively. The relative variability for both CMJ and IMTP was high to excellent (ICC: 0.88–0.98 and 0.93–0.98, respectively) in total and separately for both groups. Low measurement error (i.e., <10%) was detected for both tests and groups. In the total sample, and for male and female athletes, TE and TE% ranged between 2.2 and 2.6 cm (6.9%–8.0%) for CMJ and between 122.5 and 128.8 N (6.3%–8.9%) for IMTP. Both CMJ and IMTP showed good test sensitivity. The TE was larger than SWC_(0.2)_ but smaller than SWC_(0.5)_ except for the IMTP in female athletes, where the TE exceeded both SWC_(0.2)_ and SWC_(0.5)_, showing marginal sensitivity.

### 3.3 Discriminative validity of CMJ and IMTP

CMJ height, relative CMJ Peak Power and IMTP force were significantly different between the two groups (Table 2). The male athletes had better performance scores in CMJ height [(t-test: 3.92, p < 0.01; large ES (CI: 0.55–1.86)], relative CMJ Peak Power [(t-test: 1.80, p < 0.05; small ES (CI: 0.06-1.16)] and IMTP force [(t-test: 5.14, p < 0.01; large ES (CI: 1.13-2.57)] compared to the female athletes. However, no significant differences between the groups were identified for the CMJ Peak Power (t-test: 1.47, p = 0.07) or the relative IMTP force (t-test: 0.51, p = 0.30).

## 4 Discussion

This study is the first to investigate the reliability and validity of the CMJ and IMTP tests performed after the CPET in a heterogeneous group of elite male and female athletes. This study provides several important findings: (1) the CMJ and IMTP, performed after the CPET (i.e., a maximal exhaustion test), are reliable and sensitive measurement tests in a heterogeneous group of athletes, (2) the CMJ and IMTP tests, performed after CPET, evidence good discriminative validity by differentiating between male and female athletes.

### 4.1 Reliability of CMJ and IMTP

Previous method studies have demonstrated the relative reliability of the CMJ test (i.e., jump height) with high ICC values (i.e., >0.90) either performed on a contact and infrared mat (Markovic et al., 2004; Pojskic et al., 2015; Pojskić et al., 2014; Pojskić et al., 2015; Pojskic et al., 2018a) or a force plate (Fahey et al., 2024; Pojskić et al., 2022; Souza et al., 2020; Bright et al., 2023; Bagchi et al., 2024; Plakoutsis et al., 2023; Warr et al., 2020; Springham et al., 2024; Thomas et al., 2017). Similarly, the IMTP test has demonstrated high test-retest reliability (ICC > 0.85) when either performed on a force plate (Thomas et al., 2017; Aben et al., 2020; De Witt et al., 2018; Guppy et al., 2018) or using a load cell dynamometer (Montoro-Bombú et al., 2023; Pichardo et al., 2024; Dobbin et al., 2018; Till et al., 2018). The findings from the current study are consistent with previous studies, evidencing high to excellent relative test-retest reproducibility for the CMJ and IMTP in the total sample (ICC = 0.97 and 0.98) and separately for men (ICC = 0.88 and 0.98) and women (ICC = 0.98 and 0.93), respectively (DeVellis and Thorpe, 2021; Koo and Li, 2016). This means that when the CMJ and IMTP were performed, the participants consistently maintained their ranking order relative to others in the group, regardless of group affiliation and the fact that they performed the tests after the CPET in a fatigued state (Weir, 2005). The high relative reliability can be partially attributed to sample heterogeneity, as evidenced by considerable between-subjects variation (CV_BS_%) ranging from 17.1% to 29.2%. This considerable heterogeneity was expected, given that participants were affiliated with different sport categories (see Section 2.2, Participants), where the CMJ and IMTP tests are regularly included in performance testing in many sports, such as football and rugby, but not in sports like rowing and cycling. Logically, in heterogeneous samples, it is easier for each participant to maintain the ranking order after test-retest measurements than in homogeneous samples, thereby contributing to increased relative reliability (Weir, 2005).

Similarly, the absolute reliability of the CMJ and IMTP, evidenced by low within-subjects test-retest variability (CV_WS_%) and typical measurement error (TE%), was shown to be good, ranging between 5.7% and 6.5% and 6.3% and 8.9%, respectively (Gathercole et al., 2015; Schambra et al., 2015). The findings corroborate those of previous studies, which have shown the high absolute reliability of the CMJ (Bagchi et al., 2024; Bright et al., 2023; Markovic et al., 2004; Petré et al., 2023; Pojskić et al., 2022; Souza et al., 2020; Thomas et al., 2017) and IMTP (Dobbin et al., 2018; Montoro-Bombú et al., 2023; Pichardo et al., 2024; Till et al., 2018; Thomas et al., 2017). However, the high absolute reliability was not entirely expected, given that the CMJ and IMTP were not equally represented as measurement instruments across the sample and that the tests were performed after the CPET. It is noteworthy that the CV_WS_%, typical error, and typical error % could be more useful than the ICC, as these metrics enable comparison of reliability across various jump and isometric strength tests, regardless of calibration, measurement devices, or sample heterogeneity (Hopkins, 2000), which contrasts with the ICC, which solely provides a unitless estimate of between-subject trial differences (Weir, 2005).

Moreover, neither the CMJ nor the IMPT showed any significant systematic variation. In other words, consistent trial-to-trial differences were observed in the total sample and among the group of male athletes. Practically, this means that if the familiarisation is performed as described, two CMJ and IMTP testing trials would be sufficient (Weir, 2005). In contrast, trial-to-trial differences were observed in the group of female athletes. Specifically, the first CMJ attempt was significantly lower than the second attempt. Similarly, the first and second attempts were statistically lower than the third in the IMTP. Although all participants were required to demonstrate technical proficiency before performing the tests, the potential bias introduced by the learning effect and post-activation potentiation (PAP) should not be entirely discounted (Tanner and Gore, 2012). In brief, the learning effect refers to improvements in performance that occur simply because participants become more familiar with the test procedure, rather than due to a genuine improvement in their underlying ability or fitness level (Atkinson and Nevill, 1998; Hopkins, 2000; Tanner and Gore, 2012). The PAP refers to the acute enhancement of muscular performance following a prior high-intensity contraction (Sale, 2002; Tillin and Bishop, 2009; Pojskic et al., 2015; Pojskic et al., 2023). Logically, a longer familiarisation should be applied to avoid a confounded interpretation of the results, especially in groups of athletes who do not regularly perform the CMJ and IMTP. Furthermore, recovery time longer than 1 min should be used between test-retest trials to mitigate PAP effects.

Although none of the previous studies have investigated the reliability of the CMJ and IMTP after any exhaustive cardiopulmonary exertion activity, they have used some form of warm-up before the measurements. Specifically, the warm-up protocols before the CMJ usually consist of 5–15 min of general warm-up (i.e., low-to-moderate self-paced running or cycling), followed by 5–7 min of specific warm-up (i.e., dynamic stretching, callisthenics, sprints, jumps, *etc.*) (Bagchi et al., 2024; Bright et al., 2023; Fahey et al., 2024; Markovic et al., 2004; Plakoutsis et al., 2023; Pojskić et al., 2022; Springham et al., 2024; Warr et al., 2020). Most studies have employed 5–10 min of light-to-moderate running, dynamic stretching exercises, and a test-specific warm-up consisting of several submaximal IMTP attempts with progressively increasing intensities (e.g., 50%, 70%, 85%) (Dobbin et al., 2018; Montoro-Bombú et al., 2023; Pichardo et al., 2024; Till et al., 2018). In the current study, 15 min after the CPET, the participants performed only a specific warm-up consisting of three to four submaximal jumps prior to the CMJ and five submaximal attempts (i.e., 2% × 50%, 2% × 70%, and once 85%–90% of self-estimated maximal effort) before the IMTP. This routine proved to be an effective and sufficient way to ensure participants were familiarised with the tests, allowing for reliable results even after completing an exhaustive exercise protocol like the CPET.

In the present study, the cycle-ergometer-based CPET was used to examine VO_2peak_ by gradually increasing exercise intensity until maximal exhaustion. The high BL levels (11.3 ± 3.2 mmol/L), 5 min post-CPET, which were still at near-maximal or supramaximal exercise intensity and well above the anaerobic threshold (i.e., 4 mmol/L), evidenced the participants' exhaustion. Given that the blood lactate levels return to near-baseline (∼1–2 mmol/L) within 30–60 min after a maximal test in athletic populations (Hinojosa et al., 2021; Katoch et al., 2025), we can assume that the athletes in the current study performed both the CMJ and IMTP tests in a blood lactate-elevated state. This elevated blood lactate level could cause muscle fatigue and reduced performance due to the buildup of lactate and hydrogen (H^+^) ions, which inhibit contractile processes and weaken muscle function (i.e., muscular force production and motor control) (Theofilidis et al., 2018). In brief, H^+^ ions lower the pH, which alters the conformation of troponin and reduces the calcium (Ca^2+^) binding affinity, thereby inhibiting the exposure of actin binding sites necessary for myosin cross-bridge formation and the power stroke, the primary force-generating step in muscle contraction (Theofilidis et al., 2018; Kenney et al., 2022). This, along with reduced energy production due to a shift in pH balance and depleted energy sources (i.e., ATP, creatine phosphate, circulating glucose), could partially explain the lower results in both the CMJ and IMTP compared to previous studies performed in athletic populations (Dobbin et al., 2018; Petré et al., 2023; Pojskic et al., 2015; Pojskić et al., 2015; Pojskic et al., 2018a; Thomas et al., 2017). However, the high reliability and sensitivity of CMJ and IMTP tests following the CPET highlight their practical utility for post-exercise neuromuscular assessment in the athlete population. Their robustness even under conditions of physiological fatigue makes them potentially valuable tools for monitoring performance and recovery after exhaustive physical activities.

### 4.2 Sensitivity of CMJ and IMTP

Both the CMJ and IMTP showed good test sensitivity (i.e., SWC > typical error), which was evident by comparing SWC and typical error (Hopkins et al., 2009; Atkinson and Nevill, 1998). The typical error was larger than SWC_(0.2)_ but smaller than SWC_(0.5)_ except for the IMTP in female athletes, where the typical error exceeded both SWC_(0.2)_ and SWC_(0.5)_, showing “marginal” sensitivity. This indicates that the CMJ can be utilised to detect moderate changes that exceed 0.5 times the test’s SD, showing “good” measurement usefulness in both male and female participants (Buchheit et al., 2014; Hopkins, 2004). In practice, it means that if a participant achieves, for instance, the CMJ results of 35.3 ± 4.4 cm at baseline testing, then any post-line change above 2.2 cm (4.4 cm × 0.5) can be considered a meaningful performance change. These findings corroborate previous results of good CMJ sensitivity (SWC = 2.0 cm and typical error = 1.6 cm) in male and female basketball players (Pojskić et al., 2022). On the contrary, the IMTP showed “good” sensitivity only in the sample of male participants. Consequently, any change over time in the IMTP in the observed sample of female participants should be taken with caution. The “marginal” sensitivity could be attributed to the low experience among some female participants in performing the IMTP test and the post-activation potentiation effect, which, in turn, may increase the observed systematic measurement error (i.e., the improved test results across trials).

### 4.3 Discriminative validity

The primary performance metric of the CMJ, jump height, demonstrated a substantial discriminatory capacity to differentiate between male and female athletes, with a Cohen’s d value greater than 1.2 (Hopkins, 2004). The male athletes jumped 28.7% higher than the female athletes. Our results on CMJ height corroborate those of previous studies, which have found that men jump 24%–34% higher than women (Philpott et al., 2021; Petrovic et al., 2024; Höög and Andersson, 2021; McMahon et al., 2017; Ebben et al., 2007; Laffaye et al., 2014; Rice et al., 2017). Interestingly, the absolute peak power during the CMJ, which was higher in men, did not reach a significance level (p = 0.07), which could be attributed to the heterogeneous nature of the sample in both sex groups. This contradicts one of the previously mentioned studies, which showed higher absolute power among male basketball players compared to female players (Rice et al., 2017), but conforms with other studies that showed no differences between sexes (McMahon et al., 2017; Petrovic et al., 2024). The relative peak power (W/kg) in the present study, however, was higher in the male athletes than the female athletes, corroborating with previous studies (Márquez et al., 2017; McMahon et al., 2017; Petrovic et al., 2024; Philpott et al., 2021; Rice et al., 2017), meaning that male athletes can exert propulsive force at higher rates per kg of body mass than female athletes. The observed differences can largely be attributed to the male athletes' higher percentage of lean body mass, which is approximately 7.3%, as well as a lower body fat percentage of around 7.1%. This combination enhances their ability to generate propulsive force more effectively, allowing them to accelerate their body mass more quickly and achieve higher jump heights compared to their female counterparts (Bchini et al., 2023; Petrovic et al., 2024). This is in line with the premise that muscle volume and size of cross-sectional area could be an essential contributing factor in discriminating between male and female athletes in vertical jumping performance (Häkkinen et al., 1996; Kanehisa et al., 1994; Bchini et al., 2023). However, there are other potential discriminating factors, such as differential leg stiffness strategies, the muscle and tendon properties, and motor units’ activation level (Bojsen-Møller et al., 2005; Laffaye et al., 2014; McMahon et al., 2017), that were not examined in the present study, but contribute to advanced utilisation of stored elastic energy in the tendons and the stretch-reflex during the stretch-shortening cycle in male athletes.

It is worth noting that, although male athletes outperformed female athletes in the CMJ, the achieved results are generally lower than those reported in previous studies (Laffaye et al., 2014; McMahon et al., 2017; Petrovic et al., 2024; Philpott et al., 2021; Rice et al., 2017). This is logical, given that the participants tested in the current study were recruited from various sports where lower-body power was not a dominant physical ability (e.g., endurance and technical sports). In contrast, previous studies have been conducted in homogeneous groups of athletes where explosive power is a key performance indicator (e.g., volleyball, netball, basketball, football, and track and field sprint and jump) (Laffaye et al., 2014; McMahon et al., 2017; Petrovic et al., 2024; Philpott et al., 2021; Rice et al., 2017). Moreover, the influence of a dynamic interplay between fatigue and PAP on poorer performance results in the current study should be considered. Fatigue, resulting from CPET, could transiently impair neuromuscular function, leading to decreased peak power output and force production, while PAP could improve subsequent explosive efforts by temporarily enhancing neuromuscular performance (Sale, 2002; Tillin and Bishop, 2009). The balance between these opposing processes influences post-CPET test outcomes. Initially, fatigue may dominate, reducing performance, but as recovery progresses, PAP mechanisms may foster temporary performance gains (Collins et al., 2018). The net effect depends on the timing of measurement post-CPET, exercise intensity, and individual recovery capacity, with literature indicating that prolonged fatigue tends to suppress neuromuscular output (Cormie et al., 2011). Consequently, we can assume that a 15-min post-CPET recovery was not sufficient to favour PAP gains, but rather promoted a fatigued state (Hinojosa et al., 2021; Katoch et al., 2025), which in turn could impair the CMJ and IMTP test results.

The absolute peak force, the most frequently reported performance metric of the IMTP test, also showed great power in discriminating between male and female athletes. Specifically, the male athletes were 32.3% stronger compared to the female athletes. This finding is comparable to previous studies that investigated differences between men and women in recreational resistance-trained individuals (Merrigan et al., 2020) and athletic populations (Cornish et al., 2024; Townsend et al., 2019). However, when peak force was standardised to body mass and lean mass, the differences diminished. This corroborates the findings of Merrigan et al. (2020), who also demonstrated a higher absolute peak force in men compared to women, while the peak force relative to fat-free mass was similar between sexes. However, they showed that men exerted a higher peak force per kilogram of body mass, which contradicts our findings. The observed discrepancies in the present study can be the consequence of the heterogeneous sample, as previously described.

In general, the advanced ability of male athletes to exert absolute force and develop power can be attributed to the well-documented sex-related biological differences in body mass and lean mass. Specifically, men are, on average, taller (approximately 13 cm), heavier (approximately between 14 and 18 kg), possess more muscle mass (approximately between 18 and 22 kg), and have less fat mass (approximately between 3 and 6 kg) than the average woman (Kenney et al., 2022), which is comparable with anthropometrical data in the current study. Collectively, the findings corroborate previous performance studies, which show that the differences between the sexes in elite athletes range from 8% to 10% (de Araújo et al., 2020; Buchheit et al., 2014; Höög and Andersson, 2021; McMahon et al., 2017; Sandbakk et al., 2018).

### 4.4 Strengths and limitations

One of the key strengths of this study is the inclusion of an elite athlete sample, which enhances the relevance and applicability of the findings to high-performance sports contexts. Moreover, the cycle ergometer was used to test cardiopulmonary capacity, even though it may yield lower values of VO_2peak_ than the treadmill (Price et al., 2022; Millet et al., 2009). However, the cycle ergometer is particularly advantageous in a heterogeneous group of athletes because it provides reproducible and accurate incremental workloads, reducing injury risk and skill bias in athletes who may not be experienced runners (e.g., swimmers, rowers). Additionally, conducting the study in a strictly controlled laboratory setting ensures a high level of internal validity, allowing for precise control of variables and reducing potential confounding factors. This combination supports the reliability and specificity of the observed outcomes.

However, the study has several limitations that should be acknowledged. First, the study did not include measurements of the CMJ and IMTP tests before the CPET, which prevents a comparison of pre- and post-reliability metrics. Second, the cross-sectional research design, which included data collection at a single point in time, made it impossible to assess changes or trends over time or determine the causality of observed between-sex disparities. This could limit the ability to understand how long-term factors such as training history, hormonal fluctuations, or adaptation to sport-specific demands may influence performance differences between sexes. Third, although the equality of variances across groups was controlled, the heterogeneity of the sample could still affect the observed differences between men and women. Fourth, only the primary metrics of the CMJ and IMTP tests were analysed. Although they are highly informative and generally sufficient for assessing neuromuscular performance (e.g., power and maximal strength), they may lack the ability for a more comprehensive biomechanical evaluation and analysis.

## 5 Conclusion

In conclusion, given that the CMJ and IMTP tests demonstrated high reliability and sensitivity following CPET in a heterogeneous sample of athletes, they could be confidently used as practical tools for monitoring neuromuscular fatigue and recovery, even after exhaustive cardiopulmonary exertion activities (e.g., bycycle or running races, football or basketball matches, *etc.*). Furthermore, their stability and robustness under fatigue enhance their value in return-to-play protocols and sports science research, where valid, reproducible data are essential despite physiological stress. Their high reliability and discriminative validity support their use across sexes and diverse athlete populations for consistent performance diagnostics and longitudinal monitoring. Moreover, the findings reinforce the relevance of CMJ and IMTP as reliable, sensitive, and broadly applicable measures in both applied and research settings.

## Data Availability

The raw data supporting the conclusions of this article will be made available by the authors, without undue reservation.
